# Ovine gammaherpesvirus 2 in asymptomatic water buffaloes (*Bubalus bubalis*) from Central-western Brazil and implications for infections in buffaloes worldwide

**DOI:** 10.1007/s11250-026-04876-3

**Published:** 2026-01-27

**Authors:** Juliana Torres Tomazi Fritzen, Fellipe Danyel Cardoso Martins, Mariana Motta de Castro, Vanessa Resende Rocha Tavares, Amauri Alcindo Alfieri, Selwyn Arlington Headley

**Affiliations:** 1 Laboratory of Animal Virology, Department of Preventive Veterinary Medicine, Universidade Estadual de Londrina, Celso Garcia Cid Road, PR455 Km 380, Londrina, Paraná 86057-970 Brazil; 2 Veelab Medicina Veterinária Diagnóstica, Londrina, Paraná Brazil; 3 Programa de Pós-Graduação em Biociência Animal, Universidade de Cuiabá, Cuiabá, MT Brazil; 4 Federal Inspection Service, Ministry of Agriculture and Livestock (MAPA), Buriti Alegre, Goiás Brazil; 5 Multi-User Animal Health Laboratory (LAMSA), Department of Preventive Veterinary Medicine, Universidade Estadual de Londrina, Londrina, Paraná Brazil; 6 Laboratory of Animal Pathology, Department of Preventive Veterinary Medicine, Universidade Estadual de Londrina, Paraná, Brazil

**Keywords:** Asymptomatic buffaloes, Malignant catarrhal fever, Molecular diagnosis, Ruminant diseases, Subclinical infections

## Abstract

Ovine gammaherpesvirus 2 (OvGHV2) is the cause of sheep-associated malignant catarrhal fever (SA-MCF) in which sheep are the asymptomatic carrier-hosts with subclinical infections and/or clinical SA-MCF occurring in susceptible mammalian populations worldwide. Although SA-MCF is endemic in cattle throughout Brazil, there is only one report of OvGHV2-associated disease in water buffaloes (*Bubalus bubalis*), and comparatively few descriptions of infections by OvGHV2 in buffaloes worldwide. This study describes the molecular detection of OvGHV2 infections in asymptomatic water buffaloes and discusses the implications of infections in buffaloes worldwide. All buffaloes originated from the same farm within the State of Goiás, Central-western, Brazil, had no contact with sheep, and did not present any clinical manifestation of disease. Pulmonary, renal, and intestinal tissue fragments were randomly collected from 37 buffaloes during slaughter and used in molecular assays to detect the OvGHV2 tegument protein gene. OvGHV2 DNA was amplified from 18.9% (7/37) of all buffaloes evaluated; direct sequencing confirmed the PCR results. This report adds to the few descriptions of subclinical OvGHV2 infections in buffaloes worldwide, since most previous cases occurred in clinical outbreaks of SA-MCF. The worldwide distribution of SA-MCF and/or infections due to OvGHV2 in buffaloes may be representative of the geographical regions where this ruminant species is predominantly reared. However, subclinical infections and confusion with other similar ruminant diseases may have contributed to the comparatively reduced cases identified in buffaloes relative to cattle. Therefore, OvGHV2 associated infections may be more frequent in buffaloes worldwide than previously described.

## Introduction

Ovine gammaherpesvirus 2 (*Macavirus ovinegamma2*; OvGHV2), is a member of the genus *Macavirus*, subfamily *Gammaherpesvirinae* (ICTV 2024) and is the cause of sheep-associated malignant catarrhal fever (SA-MCF) in several mammalian species worldwide, with sheep serving as the asymptomatic carrier-hosts (Li et al. 2014; O’Toole and Li 2014). Subclinical infections associated with OvGHV2 (Headley et al. 2022; Figueiredo et al. 2025) as well as clinical SA-MCF are common within continental Brazil, with infections occurring predominantly in cattle (Headley et al. 2020), reared in most biomes of this country (Headley et al. 2024a). Additionally, special conditions, particularly the traditional simultaneous rearing of cattle and sheep on the same pastures, has resulted in the largest number of clinical outbreaks of SA-MCF in the Pampa biome of Brazil (Headley et al. 2025b). The typical clinical manifestations associated with SA-MCF in susceptible animals includes elevated fever, depression, nasal and ocular discharges, bilateral corneal opacity, lymphadenopathy, and mucosal erosions (O’Toole and Li 2014; Headley et al. 2020).

The first description of SA-MCF in buffaloes worldwide probably occurred during outbreaks in Indonesia between September 1979 and May 1982 (Hoffmann et al. 1984b). Most subsequent studies done in buffaloes were based on clinical outbreaks of SA-MCF with reports predominantly from Asia (Hoffmann et al. 1984a, b; Daniels et al. 1988a; Dharma 1988; Muthalib 1988; Damayanti et al. 1994; Wiyono et al. 1994; Teankum et al. 2006; Riaz et al. 2021), Europe (Dettwiler et al. 2011; Stahel et al. 2013; Azzam et al. 2016; Amoroso et al. 2017; Coradduzza et al. 2022), with single reports from Africa (Pfitzer et al. 2015) and the Americas (Costa et al. 2009a).

SA-MCF in cattle was initially described in the Northeastern region of Brazil in 1959 (Tokarnia et al. 1959), with subsequent outbreaks being predominant in this ruminant species (Headley et al. 2020), and more frequently in the State of Rio Grande do Sul, Southern Brazil (Headley et al. 2025b). Nevertheless, there were spontaneous outbreaks of SA-MCF in the deer (Driemeier et al. 2002; Oliveira et al. 2018), horse (Costa et al. 2009b), pig (Costa et al. 2010), and recently asymptomatic OvGHV2-related infections were described in free-ranging wild boars (Headley et al. 2024b; Headley et al. 2024d) from Brazil.

The water buffalo or Asian buffalo (*Bubalus bubalis*), has two distinct subspecies: the river (*B. bubalis bubali*s) and swamp (*B. bubalis kerebau*) buffalo, with each having distinct genetical, morphological, and physiological characteristics (Minervino et al. 2020). Although Brazil had the eighth largest population of buffaloes worldwide (Minervino et al. 2020), with approximately 1.6 million heads of buffaloes reared predominantly within the Northern region of the country (IBGE 2022), there is only one description of an outbreak SA-MCF in this ruminant species (Costa et al. 2009b). Additionally, there are comparatively few descriptions of infections by OvGHV2 in water buffaloes relative to cattle worldwide; most of these were associated with clinical outbreaks of SA-MCF (Hoffmann et al. 1984a; Costa et al. 2009b; Pfitzer et al. 2015; Amoroso et al. 2017; Coradduzza et al. 2022).

The objectives of this study were to describe the molecular findings of OvGHV2 in water buffaloes from Central-western, Brazil, and revise the existing data related to the occurrence of OvGHV2-related infections in this ruminant species worldwide.

## Materials and methods

### Animal, study location, and sample collection

These buffaloes were from the same herd located in the State of Goiás, Central-western Brazil, were approximately two years of age, and were reared primarily for beef production. Buffaloes at this farm were routinely immunized against foot and mouth disease, clostridiosis, and brucellosis. Clinical manifestations of disease syndromes were not reported. Additionally, sheep were not reared at this farm, but there were sheep herds within proximity.

Fragments of the lungs, liver, and small intestine of 37 buffaloes were randomly collected during slaughter at an abattoir under the Federal Inspection Service in the Goiás. All tissues were collected at the same moment during slaughter and maintained at -80 °C until used in molecular assays.

### DNA extraction

The tissue fragments with 1 ml of PBS were mechanically disrupted in the Tissue Lyser (Qiagen, Hilden, Germany). The samples were then centrifuged 1000 rpm for 5 min and 500 µl of supernatant were collected for DNA extraction. After pretreatment with 1% of SDS and 0.2 mg of proteinase K, nucleic acid extraction was done using a combination of the phenol/chloroform/isoamilic acid and silicon/guanidine isothiocyanate techniques (Boom et al. 1990; Alfieri et al. 2006).

### Molecular detection and sequence analysis of the OvGHV2 tegument protein gene

The extracted DNA from all tissue was submitted to a seminested PCR (snPCR) designed to amplify a 238 bp of the OvGHV2 tegument protein gene as described (Baxter et al. 1993). The amplicons were purified using Wizard^®^ SV Gel and PCR Clean-Up System (Promega Corporation, Madison, WI, USA), quantified using a Qubit Fluorometer (Invitrogen^®^ Life Technologies, Eugene, OR, USA), and then submitted to direct sequencing with an ABI3500 Genetic Analyzer sequencer with a BigDye Terminator v3.1 Cycle Sequencing Kit (Applied Biosystems, Foster City, CA, USA). All PCR products were separated by electrophoresis in 2% agarose gels, stained with ethidium bromide, and examined under ultraviolet light.

Analysis of nucleotide (nt) sequence quality and contig assembly of the OvGHV2 sequences were performed with PHRED and CAP3 homepages, respectively (http://lbi.cenargen.embrapa.br/phph/). Sequence similarity searches were performed using BLAST (http://blast.ncbi.nlm.nih.gov/). The nt sequence identity matrices were constructed using the BioEdit software version 7.0.8.0. The phylogenetic tree was reconstructed using the Neighbor-joining method with the Kimura 2-parameter model, based on 1,000 bootstrapped datasets. Only OvGHV2 strains derived from buffaloes available in GenBank were included in the phylogenetic analysis; the strain derived from this study was compared with similar strains and with the OvGHV2 prototype strain (BJ1035; GenBank accession # NC007646) using the MEGA 7 software.

### Previous descriptions of OvGHV2 infections in buffaloes worldwide

To understand the impacts of OvGHV2 on buffaloes worldwide, all previous descriptions of infections in this animal species published in English or Latin databases were located and mapped. When possibly the city and/or country in which the study was done was used as entry data to elaborate the map. Additionally, the number of buffaloes positive by any diagnostic method was used to design the Kernel map. All data obtained were then plotted on a map to understand the occurrence of this infection in buffaloes worldwide; map data with the worldwide occurrence of OvGHV2 in buffaloes was generated with the qGIS software v2.18.14.

## Results

### Molecular identification of OvGHV2 in organs of asymptomatic buffaloes

The snPCR assay detected OvGHV2 DNA in 18.9% (7/37) of the buffaloes investigated; direct sequencing confirmed these results. Infections by OvGHV2 were identified more frequently in the lungs (85.7%; 6/7), followed by the kidneys (71.4%; 5/7), and small intestine (57.1%; 4/7) of these buffaloes (Table 1). Additionally, OvGHV2 DNA was identified within all organs evaluated from three buffaloes. In comparison, OvGHV2 DNA was detected only in the lungs of three buffaloes, the kidneys of two, and in the small intestine of one buffalo.

**Table 1:** Molecular detection of OvGHV2 DNA in tissue fragments of asymptomatic buffaloes from Central-western Brazil.^1^

Buffalo #	OvGHV2 seminested-PCR		
	Lung	Kidney	Small Intestine
1	Positive	Positive	Positive
2	Positive	Positive	Positive
3	Positive	Positive	Positive
4	Negative	Positive	Negative
5	Positive	Negative	Negative
6	Negative	Positive	Negative
7	Positive	Negative	Positive

Legend. ^1^ Results are only provided from buffaloes infected with OvGHV2; data from all other buffaloes are not shown

### Phylogenetic analysis of OvGHV2 strains derived from buffaloes worldwide

The amplicons obtained from all snPCR assays during this study had 100% nt sequence identity among each other; therefore, only one strain was deposited in GenBank and included in the phylogenetic analyses. The OvGHV2 strain utilized in this analysis is referred to as OvGHV2/BR-UEL/425-GO/2021 and is deposited in GenBank (accession # PP592359).

The nt identity analysis revealed a 100% sequence homology between the strain identified during this investigation and two Italian strains (KU499857 and KU499858). Furthermore, the strain herein identified had 98.6% nt sequence identity with the prototype strain of OvGHV2 (BJ1035; GenBank # NC007646) and 96.5% nt homology with the strain identified in the brain of a buffalo (GenBank # EF199761) from Brazil. Additionally, the strain derived from this study had 98.6% sequence homology with Egyptian strains (GenBank #ON952535, ON952536, and KT725443), while there were variations between 97.9% (GenBank # MK059979 and MF977713) to 98.6% (GenBank # MK059980, MF685297, and MF685298) in nt homology with the strains identified in buffaloes from India.

**Fig. 1:**
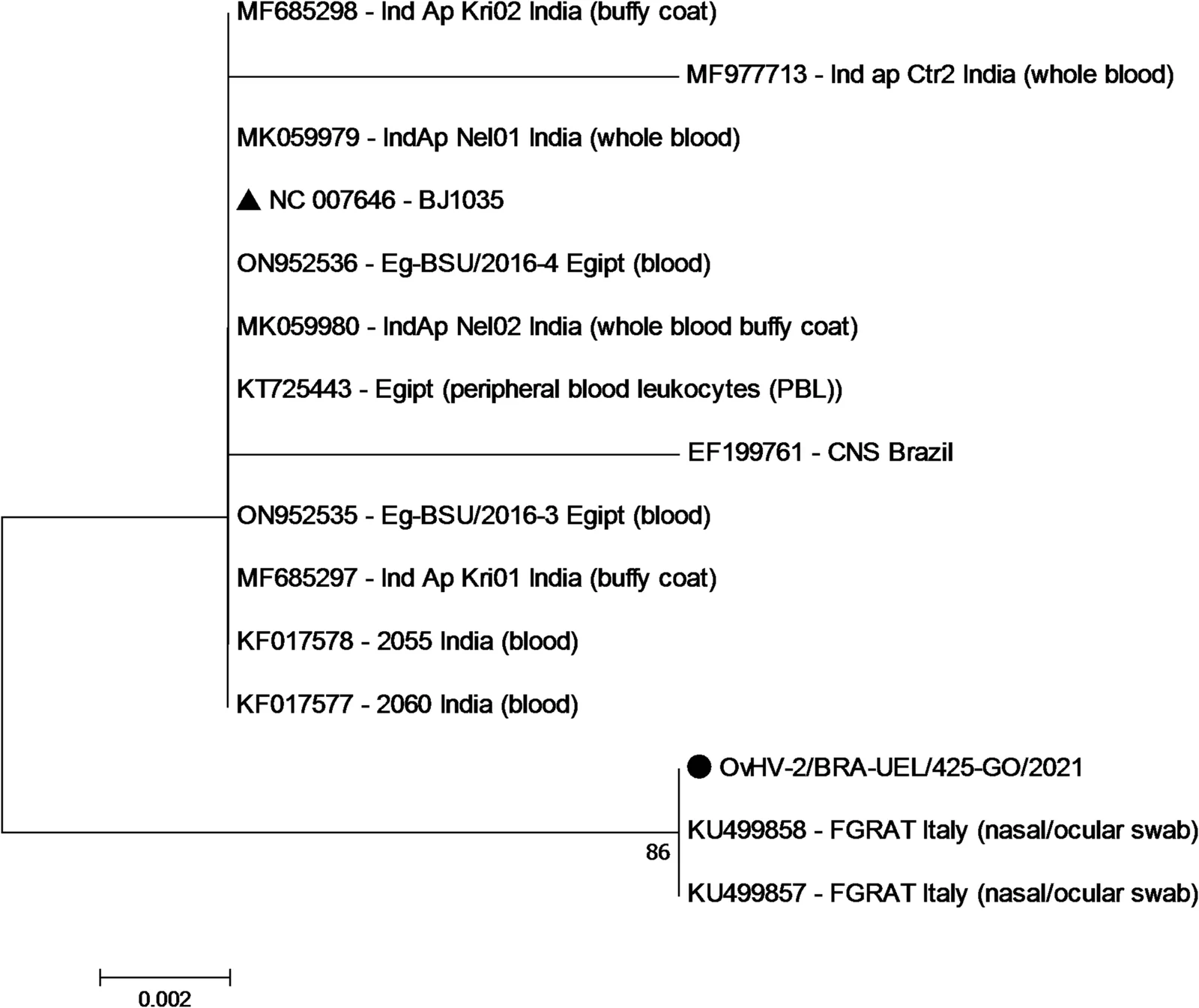
Phylogenetic tree based on the OvGHV2 tegument protein using strains derived exclusively from buffaloes. The prototype strain (BJ1035) of OvGHV2 is highlighted with a black triangle; the sequence derived from this study (●). Evolutionary history was inferred by using the Maximum likelihood method based on the Jukes-Cantor model. The strains included are identified by their GenBank accession number; the name of the strain, country of origin, and source are provided in parentheses

The phylogenetic evaluation revealed that the strain identified from this study formed a distinct cluster with strains of OvGHV2 identified in nasal and ocular swabs of buffaloes (GenBank # KU499857 and KU499858) from Italy (Fig. 1). All other strains of OvGHV2 obtained from buffaloes clustered with the reference strain (BJ1035; GenBank # NC007646).

### Worldwide occurrence of OvGHV2 in buffaloes

**Fig. 2:**
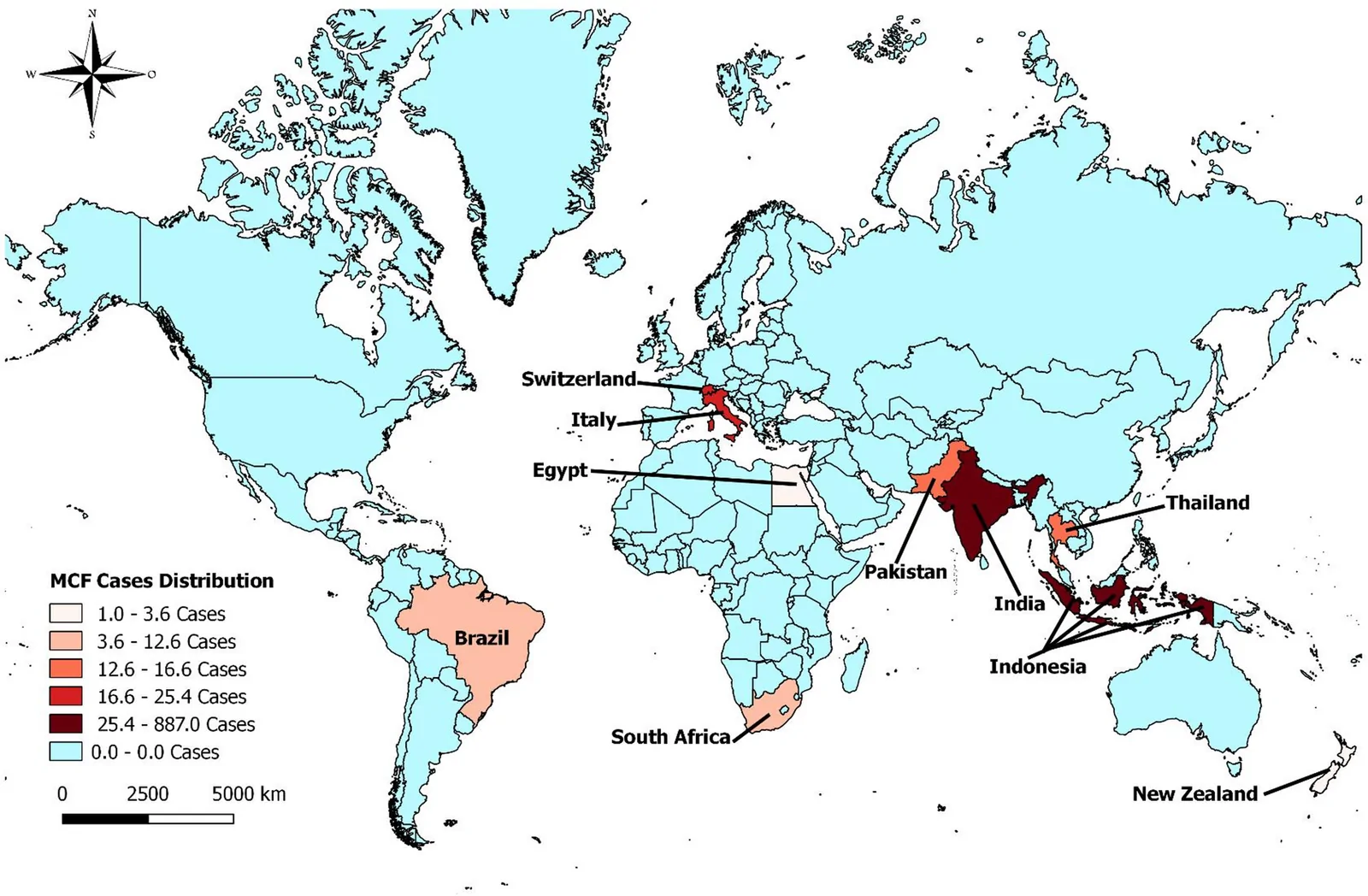
Occurrence of reported ovine gammaherpesvirus 2-associated infections in buffaloes worldwide

The distribution of published cases of OvGHV2 and/or SA-MCF in buffaloes diagnosed worldwide is presented in Fig. 2. A review of the worldwide distribution of water buffaloes (*n* = 208,098,759) revealed that these animals were reared predominantly in Asia (96.8%; *n* = 201,428,230), with India having more than half (54.8%; *n* = 114,151,770) of the estimated population of water buffaloes worldwide (Minervino et al. 2020). Accordingly, most cases of SA-MCF were identified in water buffaloes from India and Indonesia, with comparatively few cases from Nova Zealand. Additionally, it must be highlighted that the cases of MCF described in South Africa (Pfitzer et al. 2015) occurred not in water buffaloes, but in the African buffalo (*Syncerus caffer*). Curiously, Brazil was the only country in the Americas in which SA-MCF was diagnosed in buffaloes. However, the population of water buffaloes in South Africa was estimated to be less than 200 (Minervino et al. 2020), with the number of African buffaloes calculated to be approximately 121,000 (Cornelis et al. 2023), as compared to the 1.6 million heads of water buffaloes in Brazil (IBGE 2022). Therefore, it seems as if the geographical distribution of OvGHV2-induced infections in buffaloes may be representative of the worldwide population of this ruminant species.

## Discussion

During this study OvGHV2 DNA was amplified from multiple tissues of 18.9% (7/37) asymptomatic buffaloes. The detection of an infectious agent in tissues of a mammalian host without any associated clinical manifestation of disease indicates that the animal was infected (Fulton and Confer 2012). Therefore, these seven buffaloes herein described were subclinically infected by OvGHV2 or were asymptomatic. The results of this report add to the previous occurrence of SA-MCF in Brazil, considering that there was only one description of OvGHV2 in buffaloes from this country (Costa et al. 2009a). Additionally, these findings represent the second description of OvGHV2 associated infections in buffaloes from the Americas. However, in the previous report of buffaloes from Brazil, the affected animals developed typical clinical manifestations of SA-MCF and were reared concomitantly with sheep on the same farm (Costa et al. 2009a). Alternatively, in the current study, there was no known comingling between the subclinically infected buffaloes and the asymptomatic carrier-hosts (sheep) of OvGHV2; similar findings were described in a study from Switzerland (Stahel et al. 2013). Therefore, infections in buffaloes due to OvGHV2 may occur without close contact with sheep (see below).

The phylogenetic analyses revealed that the OvGHV2 strain herein identified had between 96 and 100% nt sequence homology with the OvGHV2 reference strain and with strains identified in buffaloes worldwide. Similar findings were described (Headley et al. 2015, 2024d); these results demonstrated that the OvGHV2 tegument protein gene is well conserved within all animal species (Dunowska et al. 2001). Therefore, this gene should only be used for the diagnosis of OvGHV2, while the evaluation of specific loci of ORF50 and ORF75 may provide information related to genetic polymorphism in OvGHV2 strains circulating within mammalian populations worldwide (Russell et al. 2014).

### Subclinical related-OvGHV2 infections contribute to reduced diagnosis worldwide

As stated above, the buffaloes herein described were subclinically infected by OvGHV2; similar findings were previously described in buffaloes (Stahel et al. 2013; Riaz et al. 2021), cattle (Li et al. 2001; Powers et al. 2005; Stahel et al. 2013; Martins et al. 2017; Headley et al. 2022, 2023; Figueiredo et al. 2025; Headley et al. 2025a), the American bison (Li et al. 2001; O’Toole et al. 2002; Sausker and Dyer 2002), and wild boars (Headley et al. 2024b; Headley et al. 2024d). The ever-increasing number of subclinical OvGHV2 infections in mammalians may have several interpretations: (a) infections by OvGHV2 are probably more widespread than previously documented; (b) the typical clinical manifestations associated with SA-MCF such as oral ulcerations with consequent profuse salivation could have been misdiagnosed as bovine viral diarrhea or other similar diseases; or (c) the disease is probably neglected.

Collectively, this then indicates that SA-MCF and/or infections by OvGHV2 are probably underdiagnosed worldwide, as was previously suggested (Headley et al. 2020). Additional factors that could have contributed towards the misdiagnosis or underdiagnosis of SA-MCF are the histological absence of necrotizing vasculitis or fibrinoid change to the affected arteries in cattle with clinical manifestations attributed to this disease. Although these histological vascular alterations are the hallmarks of SA-MCF (Li et al. 2014; O’Toole and Li 2014; Headley et al. 2020), these arterial lesions are progressive and may terminate in vascular obliteration, referred to as arteriopathy (O’Toole et al. 2002) or progressive vascular lesions (Headley et al. 2022). Consequently, necrotizing lymphocytic vasculitis or fibrinoid change may not always be present during histological evaluation, resulting in a non-diagnosis of OvGHV2-related infection and/or SA-MCF. This was demonstrated in cattle with chronic (O’Toole et al. 1995) and subclinical infections (Headley et al. 2022; Headley et al. 2025a) due to OvGHV2, as well as in subclinically infected American bison (Schultheiss et al. 1998), during which typical vascular lesions were not observed in these animals.

Moreover, the occurrence of clinical SA-MCF or subclinical OvGHV2-related infections may be influenced the following: (a) the pathogenicity of the specific strains of virus circulating within a specific geographical; (b) the number of viral particles shed by sheep with the potential to infect populations of ruminants (Russell et al. 2014); (c) the distinct husbandry and environmental conditions within a specific geographical region (Russell et al. 2014; Headley et al. 2024a, d) the proportion of sheep reared concomitantly with the susceptible mammalian species (Headley et al. 2025b), and e) specific herd genetics that favor virus transmission (Russell et al. 2014). Consequently, the detection of OvGHV2-related infections in buffaloes and other mammalians may be more complicated in the absence of typical gross and/or histopathological findings.

During this study, 18.9% (7/37) of the tissues from the buffaloes evaluated were subclinically infected by OvGHV2; this frequency of detection was more elevated than the 16.2%% (24/148) identified in blood samples from asymptomatic buffaloes from Switzerland (Stahel et al. 2013), but less than the 26% (13/50) described in blood samples from buffaloes with similar health status from Pakistan (Riaz et al. 2021). The identification of OvGHV2 in the blood of subclinically infected buffaloes from Switzerland (Stahel et al. 2013) and Pakistan (Riaz et al. 2021), suggest that these infected buffaloes were viremic. Similarly, OvGHV2 was detected in the blood of asymptomatic American bison (Schultheiss et al. 1998, 2000), and in the lungs of cattle with interstitial pneumonia from Mato Grosso, Brazil (Figueiredo et al. 2025). Additionally, an American bison subclinically infected with OvGHV2 was reportedly without vascular alterations sufficient to develop clinical disease for at least two months (Schultheiss et al. 1998), demonstrating that subclinically infected ruminants can maintain the virus for prolonged periods without clinical evidence of disease. However, during the current study blood samples were not collecetd due to routine dynamics of the slaughterhouse, so it is unknown if these buffaloes were viremic.

Accordingly, the frequent diagnosis of subclinical OvGHV2-related infections in mammalians from Brazil may suggest that the real prevalence of OvGHV2 in this continental nation is more elevated than previously described. This may also be applied to the identification of OvGHV2 infections worldwide, and was corroborated with the demonstration of OvGHV2 antibodies in 7.9% (29/367) of cows maintained on 37.2% (16/43) of closed dairy farms without any contact with sheep from Southern Brazil (Headley et al. 2024c). Consequently, additional seroepidemiological surveys are being developed to determine the prevalence of OvGHV2 in selected ruminant populations from distinct geographical regions and biomes of Brazil.

### Overview of OvGHV2 infections in buffaloes worldwide

The large number of buffaloes diagnosed in Indonesia probably reflects the result of an international meeting done in that country that was based essentially on SA-MCF in buffaloes (Daniels et al. 1988b), since the number of buffaloes in Indonesia was comparatively less to that of India, where more than half of the worldwide population of buffaloes were reared (Minervino et al. 2020).

In the current study, all buffaloes were subclinically infected. Nevertheless, the clinical and pathological manifestations of SA-MCF observed in buffaloes are quite similar to those observed in cattle (Martínez-Burnes et al. 2024), with the “head and eye” form being most frequently diagnosed. However, disseminated vasculitis within the myometrium (Teankum et al. 2006; Azzam et al. 2016) and ovarian vessels (Teankum et al. 2006) seemed to be a prominent feature of this disease in buffaloes. These severe vascular lesions within the female reproductive tract may explain the molecular detection of OvGHV2 in the fetus of a buffalo from Switzerland (Stahel et al. 2013), confirming vertical transmission. Collectively, these findings suggest that vertical infection due to OvGHV2 as described in cattle (Headley et al. 2015; Rosato et al. 2021; Silva et al. 2024), sheep (Headley et al. 2025c), and bison (Schultheiss et al. 1998), may also be a form of transmission in buffaloes.

Geographically, water buffalos are reared predominantly in Asia (Minervino et al. 2020; Zhang et al. 2020), with approximately 69% of the river buffaloes in India and 63% of swamp buffaloes from China (Zhang et al. 2020). Paradoxically, there are few descriptions of OvGHV2 infections in buffaloes from India (Singh et al. 1979), while reports were was identified from China. Therefore, one wonders if the extremely reduced number of reports of OvGHV2-related infections in these countries with the largest populations of buffaloes is directly related to underdiagnosis due to subclinical infections, misdiagnosis due to possible confusion with similar ruminant diseases or whether these animals are more resistant to infection by OvGHV2 than previously considered. The absence of a diagnosis of SA-MCF in ruminants over a 30-year period in India was related to possible confusion with ruminant diseases including infectious bovine rhinotracheitis, vesicular stomatitis, and the bovine viral diarrhea-mucosal disease complex (Sood et al. 2013). Consequently, underdiagnosis may be associated with the comparatively reduced number of reports of SA-MCF in buffaloes as compared to cattle.

In most outbreaks of SA-MCF in buffaloes, the affected animals intermingled with sheep (Singh et al. 1979; Costa et al. 2009a; Dettwiler et al. 2011; Amoroso et al. 2017; Coradduzza et al. 2022) or were within proximity to sheep (Hoffmann et al. 1984b; Azzam et al. 2016). However, during this investigation there was no contact between the subclinically infected buffaloes and sheep; similar findings were described on a farm in Switzerland, where the possibility of buffalo-to-buffalo infections was considered (Stahel et al. 2013). Alternatively, the source of infection remains obscure in the current cases. It must be highlighted, that there were outbreaks of SA-MCF in cattle reared in several geographical regions of Brazil without any contact with the asymptomatic carrier host (Corrêa et al. 1972; Lemos et al. 2005; Headley et al. 2013). These findings may suggest that there may be another animal species that can serve as some form of intermediate or bridge host for the dissemination of OvGHV2 to susceptible ruminant populations in this continental nation (Headley et al. 2024b). Interestingly, the presence of free-ranging wild boars (*Sus scrofa*) subclinically infected by OvGHV2 (Headley et al. 2024b) was associated with the development of cutaneous infections (Headley et al. 2025d) and the serological detection of OvGHV2 antibodies (Headley et al. 2024c) in dairy cattle from Southern Brazil that had no prior contact with neither sheep nor goats. Similar findings were observed in bovine neonates that were transplacentally infected by OvGHV2 (S.A. Headley, personal communications). Accordingly, free-ranging wild boars may serve as potential bridge-hosts for the transmission of OvGHV2 to susceptible mammalian populations in geographical regions where sheep are not reared concomitantly with cattle (Headley et al. 2024c). Alternatively, special wind conditions and the possibility of viral dissemination by birds were proposed as possible explanations for OvGHV2 infections in bison that were reared 5 km distant from sheep (Li et al. 2008). Therefore, there are still grey areas that must be elucidated so that the epidemiology and pathogenesis of SA-MCF or infections by OvGHV2 can be totally elucidated.

### Study limitations and future perspectives

The simultaneous collection of blood samples from all buffaloes would have demonstrated if these subclinically infected animals were viremic at the time of collection. However, during routine *post-mortem* inspection there are distinct examination lines that make the simultaneous obtention of blood and associated tissues from the same animal difficult without causing disarray within the abattoir. Nevertheless, these findings provide additional insights related to the understanding of this disease in buffaloes.

Another limitation was the non-collecting of tissue samples for histopathological evaluation and the possible evaluation of buffaloes over a longer period. Histopathological analyses would have been fundamental to determine if the infected buffaloes were diseased, since the detection of an infectious disease agent in animal tissues with typical histological alterations indicates disease (Fulton and Confer 2012). The analysis of buffalo populations over longer periods and with a larger number of samples would have been useful to determine possible patterns associated with infections.

Accordingly, studies are being implemented, in conjunction with other research institutes of Brazil, to evaluate the occurrence of OvGHV2-related infections in buffaloes from different geographical regions of this country so that the real distribution of this infection within this ruminant species can be estimated. Additionally, phylogenetic analyses using specific loci of OvGHV2 are in progress to compare possible genetic differences between the strains circulating within distinct biomes of Brazil and between distinct geographical locations.

## Conclusion

Ovine gammaherpesvirus 2 DNA was detected in multiple organs of 18.9% (7/37) buffaloes without clinical manifestations of disease, demonstrating that these animals were subclinically infected. Additionally, these subclinically infected buffaloes had no contact with sheep. The detection of subclinical infection in buffaloes from this geographical location of Brazil adds to the number of cases of SA-MCF without prior exposure to sheep as well as to the rising number of OvGHV2-related subclinical infections worldwide. The occurrence of subclinical infections due to OvGHV2 and clinical SA-MCF in buffaloes worldwide is probably underdiagnosed due to misdiagnosis or confusion with similar ruminant diseases. Therefore, the number of buffaloes infected by this pathogen is probably more elevated than previously reported.

## Data Availability

The datasets generated during and/or analyzed during the current study are available in the GenBank, under accession number PP592359, available at https://www.ncbi.nlm.nih.gov/nucleotide/.
